# The clinical usefulness of a web-based messaging system between patients with Crohn disease and their physicians

**DOI:** 10.1097/MD.0000000000004028

**Published:** 2016-07-01

**Authors:** Da Eun Jeong, Kyeong Ok Kim, Byung Ik Jang, Eun Young Kim, Jin Tae Jung, Seong Woo Jeon, Hyun Seok Lee, Eun Soo Kim, Kyung Sik Park, Kwang Bum Cho, Daegu-Gyeongbuk Gastrointestinal Study Group (DGSG)

**Affiliations:** a Division of Gastroenterology and Hepatology, Department of Internal Medicine, Yeungnam University College of Medicine, Daegu, Korea; b Catholic University of Daegu School of Medicine, Daegu, Korea; c Kyungpook National University College of Medicine, Daegu, Korea; d Keimyung University College of Medicine, Daegu, Korea.

**Keywords:** Crohn disease, messages, web-based system

## Abstract

To avoid missing events associated with clinical activity, the authors previously developed a novel, web-based, self-reporting Crohn disease (CD) symptom diary. However, although this diary provided a means of self-checking based on responses to set questions based on Harvey–Bradshaw index scores, it was limited in terms of describing other specific symptoms. Thus, the authors added a space to the questionnaire, which allows patients to send clinicians questions or a description of unpredictable events. The aim of the present study was to assess the clinical usefulness of this messaging system by analyzing patients’ messages.

The messaging system between patients and their doctors was included in a webpage created for recording patients’ symptom diaries (www.cdsd.or.kr). Using this system, patients can send messages easily at any time and doctors can read and respond to these messages immediately using a smart phone or computer. In the present study, the authors retrospectively reviewed 686 messages sent by 152 patients from July 2012 to July 2014 and patient medical records.

Mean patient age was 29.0 ± 11.6 years and the male-to-female ratio was 99:53. Most messages regarded symptoms (381 messages, 55.5%), which was followed by self-reports about general condition (195 messages, 28.4%) and questions about treatment (71 messages, 10.3%). With respect to symptoms, abdominal pain was most common (145 cases, 21.1%) followed by hematochezia (36 cases, 5.2%). Problems about medication were the most frequently associated with treatment (65, 91.5%). Patients above 40 years showed a greater tendency to focus on symptoms and treatment (*P* = 0.025). The doctor answer rate was 56.3% (n = 386), and based on these responses, an early visit was needed in 28 cases (7.3%).

Using this web-based messaging system, patients were able to obtain proper advice from their physicians without visiting clinics or searching the Internet, and in addition, 7.3% of messages prompted an early visit. Although longer follow-up is required, this study shows that the devised messaging system provides a clinically relevant communication tool for patients and physicians.

## 1 Introduction

Recently, interest in web-based messaging systems for patients with chronic diseases has increased. Crohn disease (CD) is a chronic inflammatory disease that needs lifelong constant care and treatment.^[1,2]^ We previously described a self-reporting CD symptom diary (CDSD) that was devised to document patient condition between visits and to aid in the prediction of disease course.^[3]^

When patients cannot visit to resolve some unexpected problem, they tend to ask other patients questions on the web. Often patients exchange useful information via Crohn disease sites, but sometimes inaccurate information is provided. For this reason, we provided a messaging facility on a pre-existing diary system that can be used to upload details of patient status and to communicate with doctors. When appropriately used, this messaging system allows patients to consult their doctor directly and obtain answers to problems, which could be highly beneficial for patients and doctors.

In this study, we evaluated the clinical usefulness of the devised messaging system by analyzing complaints and questions put to doctors.

## 2 Methods

**Figure 1:**
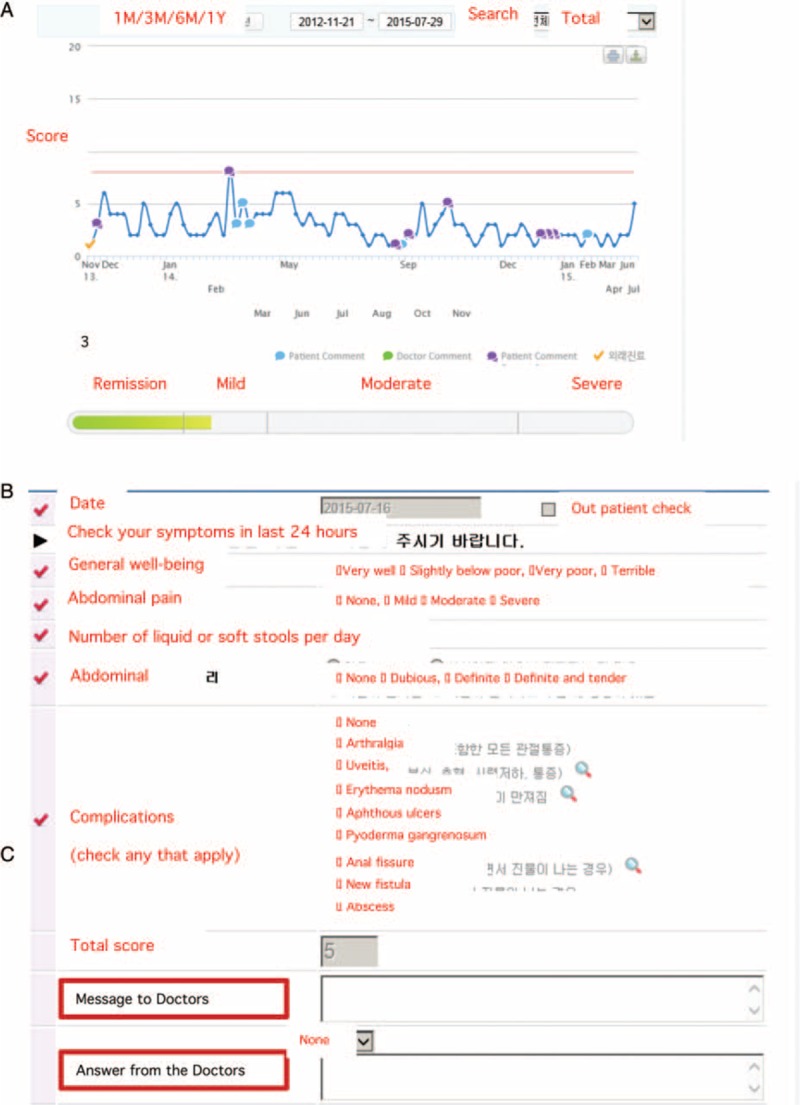
Crohn disease symptoms diary (CDSD). A = Time plot of Harvey–Bradshaw index (HBI) scores. B = Click box for entering total HBI score. C = Message system click box. ^∗^In the actual system, all items are in Korean. M = month, Y = year.

We opened a messaging space between patients and doctors in the web page previously prepared for recording the symptom diary (www.cdsd.or.kr). This diary addresses 5 clinical parameters based on the Harvey–Bradshaw index, that is, general well-being, abdominal pain, number of liquid stools per day, abdominal mass, and presence of CD-associated complications, which are awarded a score of 0 or 1. These scores are then totaled and entered into a click box by the patient, and the total is automatically added to the time plot (Fig. 1A and B).

Messages sent by patients can be responded to immediately by phone or computer (Fig. 1C). For facilitate real-time communication, doctors are notified of incoming messages using an alarm. In the present study, we retrospectively reviewed 686 messages from 152 patients and patient medical records from July 2012 to July 2014. All patients provided informed consent and could see only their data which are considered to be the most personal and sensitive information. The messaging system involves the use of IDs and passwords, and thus, only authorized physicians can access patient data. According to recent studies on the e-health system, privacy protection is essential.^[4]^ Authorized access, data encryption, patient consent, and monitoring of system access are required to maintain the security and privacy of an e-health record.^[5]^ Accordingly, the ethics review committees of the Institutional Review Boards regularly monitor the security of data systems.

To analyze patient baseline characteristics and clinical features, messages were classified as symptom, treatment, prognosis, condition, food, or pregnancy/delivery related or others. Symptoms were subdivided into abdominal pain, bleeding, diarrhea, fistula, fever, nausea/vomiting, and others. Treatment was subdivided into medical treatment (drug information, side effects), surgery, and others. We also investigated physician response rates and early admissions resulting from messages. Continuous variable such as age and disease duration was described as mean + SD. This study was approved by our institutional review board (2012-01-460).

## 3 Results

The mean age of the 152 CD patients was 29.0 ± 11.6 years and the male-to-female ratio was 99:53 (Table 1). Mean disease duration was 56.9 ± 48.1 months. Ileocolic involvement was the most common (78, 51.3%) followed by small bowel involvement (40 cases, 26.4 %). In more than half of the patients the disease was inflammatory (92, 62.5%), and in the others it was stricturing (29, 19.1%), or penetrating (28, 18.4%). Eighty-three patients (54.6%) had a history of surgery. 5-aminosalicylate was used in 145 (95.4%) and azathioprine/6-mercaptopurine in 127 (83.6%). Eighty-one patients (53.3%) were students and 38 (25.0%) were office workers.

**Table 1:**
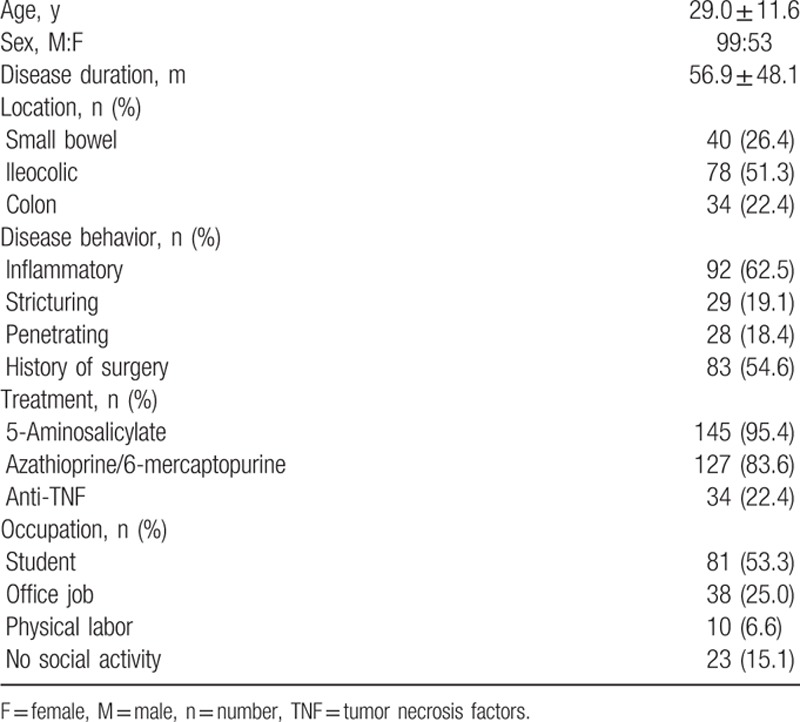
Clinical characteristics of Crohn disease patients.

**Figure 2:**
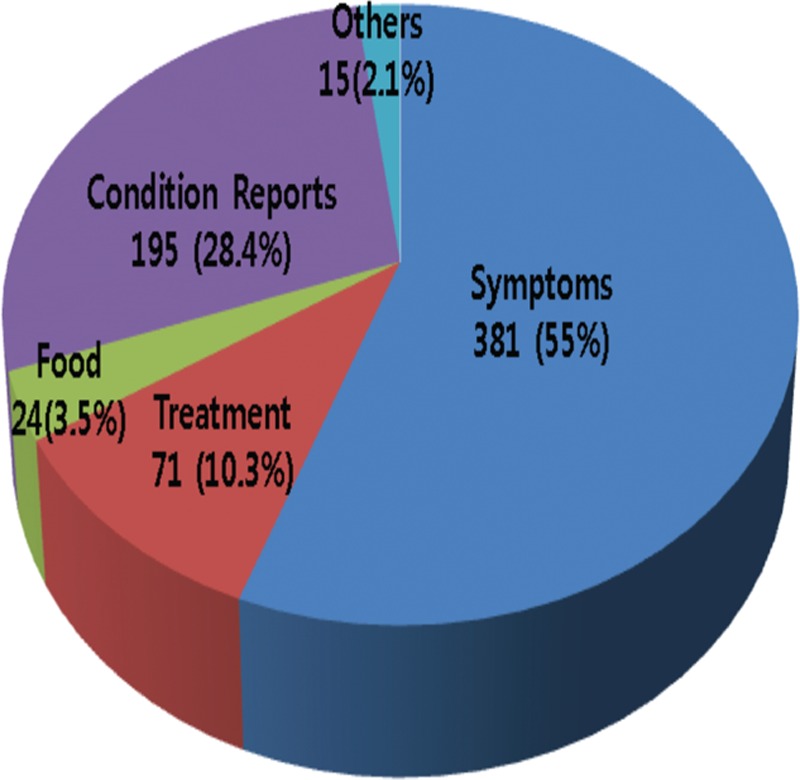
Frequencies of question topics.

**Figure 3:**
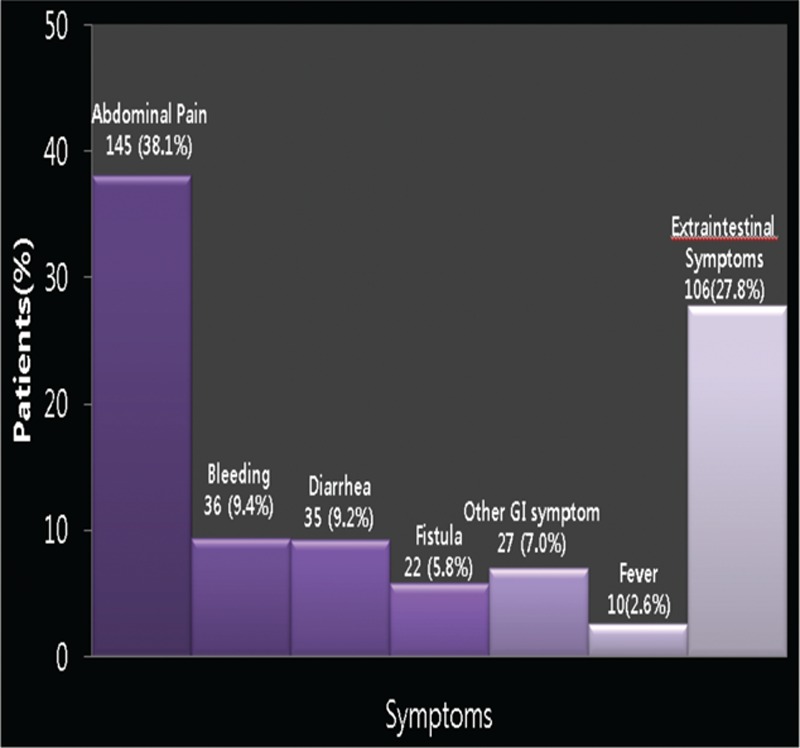
Distribution of questions regarding symptoms.

The mean number of messages sent per patient over the 24 months study period was 4.5. Messages most commonly involved symptoms (381, 55.5%) followed by treatment (71, 10.3%) (Fig. 2). Abdominal pain was the most common problem (145 messages, 21.1%) followed by hematochezia (36, 5.2%). Other than abdominal symptoms accounted for 106 messages (27.8%) (Fig. 3). Problems about medication were the most frequent subjects of messages associated with treatment (65, 91.5%). Patients aged under 40 years sent more variable messages, whereas patients above 40 years tended to focus on symptoms and treatment (*P* = 0.025). However, question types were not found to depend on disease extent or behavior.

The answer rate by doctors was 56.3% and an early visit (based on physicians’ responses) was deemed necessary after 28 messages (7.3%). Furthermore, 163 patients (42.2%) who received responses from physicians improved without visiting a clinic (Table 2).

**Table 2:**
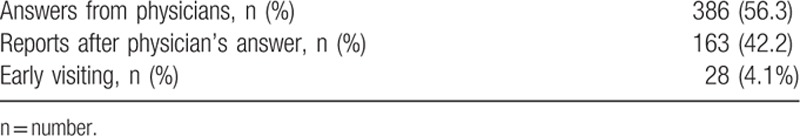
Outcomes of messages.

## 4 Discussion

Because of the convenience and effectiveness of the e-health system, many studies have been undertaken to assess the use of the web and mobile services for chronic disease management such as hypertension,^[6]^ diabetes mellitus,^[7–10]^ and chronic heart failure.^[11]^ In diabetes mellitus, several studies reported that the telemanagement system was found to reduce glycated hemoglobin levels by coaching lifestyle behaviors using patient self-management data.^[8–10]^

A web-based telemanagement system also has been introduced for patients with inflammatory bowel disease (IBD) that encourages active patient involvement in the disease assessment and treatment.^[12–15]^ In addition, Litcher-Kelly et al reported a high rate of IBD patient compliance (88%) using software installed on a handheld computer.^[16]^ In another study on infliximab treatment using a web-based approach in CD, it was suggested that the technique offered a novel means of individualizing treatment. However, the study was limited to 27 selected patients.^[13]^

The majority of patients tend to resolve problems using the internet or by asking other patients. Many web sites have been set up to allow patients to discuss problems, but relatively few however offer the support of a physician, and as a result sometimes patients are misinformed. Furthermore, although a self-managed, web-based, monitoring system was recently developed for CD patients, it does not incorporate a messaging system but rather uses close-ended questions.^[12,13,16,17]^

Our Daegu-Gyeongbuk Gastrointestinal Study Group (DGSG) group created a messaging space in a previous CD symptom diary site (www.cdsd.or.kr) for physicians and patients. However, although patients sometimes receive good responses from individuals with the same disease, they are occasionally misinformed, especially when faced with some emergent problem, to the extent that outcomes could have been jeopardized. Although the best way to solving an unexpected problem is to visit a physician, sometimes this is not possible. Furthermore, patients are not prepared to visit physicians to have what may be trivial questions answered, and physicians have insufficient time to answer question fully in an outpatient setting. In our opinion messaging systems, similar to that described, could solve these problems. The messaging system we have devised allows patients to ask open-ended questions without limitation. This technology also helps build good relationships between patients and physicians. In fact, in the present study, 28.4% of messages were useful patient reports following previous communication with a physician. Furthermore, the web and mobile phones are accessible worldwide, and offer an excellent means of communication patients and physicians on a huge scale. More than half of the patients involved in the present study were students and a quarter were office workers, and thus, our study subjects were familiar with internet services and found it easy to access our message system and the electronic diary. The limitation of the message system is that there is no alarm service for the patients when physician sends an answer for their question. Therefore, the patient should check into the app directly. However, the patients send a message when they are in difficult condition, they wait for answers from their physician. In that reason, patients frequently come to the app and the possibility of missing early visiting recommendation or inappropriate actions is very low. Actually, there were no reported cases of inappropriate action due to delayed checking of answers. We are now trying to improve the system for this point.

Our message analysis showed sudden symptom development was the most common concern, and as a direct result of using the system several patients made unscheduled visits for appropriate treatment, which indicates if managed well such a system could be used to detect and prevent disease exacerbations. In the present study, the doctor answer rate was 56.3%, but it should be borne in mind that 28.4% of messages were status reports, and thus, the actual response rate was 78.6%.

To establish a web-based management system and improve the quality of medical care, doctors must provide convincing answers. Some doctors appear to be concerned that patients might abuse such systems, but no evidence of this was found in the present study, and no nondisease-related question was asked of physicians.

Summarizing, using our novel messaging system, patients with Crohn disease were able to ask doctors questions about emergent problems, such as symptoms, and get appropriate direct advice without visiting a clinic or searching the Internet. Furthermore, the study shows that 7.3% of messages resulted in an unscheduled hospital visit. Additional long-term studies are required, but results to date show that the devised web-based messaging system offers patients and physicians a very useful communication tool.
